# Structural Optimization and De Novo Design of Dengue Virus Entry Inhibitory Peptides

**DOI:** 10.1371/journal.pntd.0000721

**Published:** 2010-06-22

**Authors:** Joshua M. Costin, Ekachai Jenwitheesuk, Shee-Mei Lok, Elizabeth Hunsperger, Kelly A. Conrads, Krystal A. Fontaine, Craig R. Rees, Michael G. Rossmann, Sharon Isern, Ram Samudrala, Scott F. Michael

**Affiliations:** 1 Department of Biological Sciences, Florida Gulf Coast University, Fort Myers, Florida, United States of America; 2 Department of Microbiology, University of Washington, Seattle, Washington, United States of America; 3 Department of Biological Sciences, Purdue University, West Lafayette, Indiana, United States of America; 4 Dengue Branch, Division of Vector-Borne Infectious Diseases, Centers for Disease Control and Prevention, San Juan, Puerto Rico; 5 FortéBio, Incorporated, Menlo Park, California, United States of America; University of California, Berkeley, United States of America

## Abstract

Viral fusogenic envelope proteins are important targets for the development of inhibitors of viral entry. We report an approach for the computational design of peptide inhibitors of the dengue 2 virus (DENV-2) envelope (E) protein using high-resolution structural data from a pre-entry dimeric form of the protein. By using predictive strategies together with computational optimization of binding “pseudoenergies”, we were able to design multiple peptide sequences that showed low micromolar viral entry inhibitory activity. The two most active peptides, DN57opt and 1OAN1, were designed to displace regions in the domain II hinge, and the first domain I/domain II beta sheet connection, respectively, and show fifty percent inhibitory concentrations of 8 and 7 µM respectively in a focus forming unit assay. The antiviral peptides were shown to interfere with virus:cell binding, interact directly with the E proteins and also cause changes to the viral surface using biolayer interferometry and cryo-electron microscopy, respectively. These peptides may be useful for characterization of intermediate states in the membrane fusion process, investigation of DENV receptor molecules, and as lead compounds for drug discovery.

## Author Summary

Virus surface proteins mediate interactions with target cells during the initial events in the process of infection. Inhibiting these proteins is therefore a major target for the development of antiviral drugs. However, there are a very large number of different viruses, each with their own distinct surface proteins and, with just a few exceptions, it is not clear how to build novel molecules to inhibit them. Here we applied a computational binding optimization strategy to an atomic resolution structure of dengue virus serotype 2 envelope protein to generate peptide sequences that should interact strongly with this protein. We picked dengue virus as a target because it is the causative agent for the most important mosquito transmitted viral disease. Out of a small number of candidates designed and tested, we identified two different highly inhibitory peptides. To verify our results, we showed that these peptides block virus:cell binding, interfere with a step during viral entry, alter the surface structure of dengue viral particles, and that they interact directly with dengue virus envelope protein. We expect that our approach may be generally applicable to other viral surface proteins where a high resolution structure is available.

## Introduction

Fusogenic viral envelope glycoproteins are multimeric proteins that facilitate the fusion of viral and target cell lipid membranes during the initiation of infection. The membrane fusion process is energetically favorable and essentially irreversible, but has a considerable kinetic energy barrier [1]. These proteins allow rapid membrane fusion by drawing the opposing membranes together and either stabilizing or providing the activation energy to surmount the transition state [1], [2]. In this way, they behave in many aspects like a fusion catalyst. Because they effect a macromolecular process that involves large scale conformational changes in the substrate membranes and the proteins themselves, these proteins possess multiple interacting surfaces that could be targeted by inhibitors [3].

There are several distinct types of viral fusion proteins, including the class I, primarily alpha helical proteins (such as HIV TM and influenza HA), the class II, primarily beta sheet proteins (such as the flavivirus E and alphavirus E1), and mixed helix/sheet proteins (including herpes virus gB and rhabdovirus G) [3], [4]. To date, most progress with viral fusion protein inhibitors has focused on class I alpha helical proteins. The HIV TM protein provides an excellent example of targeting distinct, interacting surfaces for inhibition. The HIV TM functions as a homotrimer with each monomer contributing two alpha helical regions that interact to form a post-fusion six-helix bundle. Inhibition of the formation of this six-helix bundle can be accomplished by exogenous peptides mimicking either of the two reciprocally interacting helices [5]–[7].

Only a few examples of viral entry inhibitors with activity against the primarily beta sheet envelope proteins (E) from flaviviruses have been described [8]–[10]. However, few of these have taken advantage of the available crystal structures of flavivirus E proteins, including both pre-fusion and post-fusion forms [11]–[22]. The authors of some of these structures have predicted that several regions of these proteins might be targets for inhibition [11], [14], [15]. Here we report the use of structural data from the pre-fusion dengue virus-2 (DENV-2) E protein as a model for a computational approach to the design of new peptide inhibitors of DENV-2 entry. This approach makes use of a residue-specific all-atom probability discriminatory function (RAPDF) score to identify *in situ* amino acid sequences that are likely to have high structural and binding stability [23], [24]. Out of seven computationally designed peptides that were synthesized and tested, two were identified as possessing fifty percent *in vitro* inhibitory activity (IC_50_) below 10 µM and another with IC_50_ activity below 40 µM. Two of the inhibitors (DN57opt and DN81opt) are binding optimized variants of peptides originally designed from DENV inhibitory peptide sequences located in domain II near the domain I/domain II hinge region [9]. The other (1OAN1) is an entirely novel peptide designed from an extended beta sheet region comprising the first connection between domains I and II. We show that the two peptides with the highest inhibitory activity interfere with virus:cell binding, cause structural changes to the surface of DENV-2 virions, and bind specifically to purified DENV-2 E protein.

The causative agent of dengue fever, dengue hemorrhagic fever and dengue shock syndrome, DENV has emerged in the past several decades as the most important mosquito borne viral disease with an estimated 2.5 billion people living in areas at risk for epidemic transmission and 50–100 million people infected annually [25], [26]. Complicating this situation, the four distinct serotypes of DENV generate only low level immunological cross protection, allowing for repeated epidemic outbreaks in the same populations [27], [28]. The phenomenon of antibody dependent enhancement has been shown to result in more severe disease in individuals who have been previously infected with a different serotype [29]–[33]. With no specific treatment or prevention available other than vector control, DENV is an important target for the development of antivirals and vaccines. The results presented here indicate that the DENV E glycoprotein has multiple accessible surfaces that can be targeted by distinct inhibitors and is an amenable target for rational inhibitor design.

## Materials and Methods

### Computational optimization of hinge region inhibitory peptides

Peptide inhibitors were designed to have improved in situ binding compared to naturally occurring sequences using the residue-specific all-atom probability discriminatory function (RAPDF) [24]. The x-ray diffraction structure of DENV-2 envelope protein (Protein Data Bank identifier 1OAN) was used as a template for creating mutant structures from which the peptides were derived [14]. For each peptide, we randomly selected a residue side chain and substituted it with a new side chain. The substitution was performed using a backbone-dependent side chain rotamer library and a linear repulsive steric energy term provided by SCWRL version 3.0 [34]. The resulting all-atom models were energy minimized for 200 steps using the Energy Calculation and Dynamics (ENCAD) program [35]–[37]. RAPDF scores were then calculated to estimate the structural stability of a given E protein structure derivative. For a selected residue, side chain substitution was carried out ten times. The amino acid that produced the best RAPDF score was selected and used as a template for further mutation. The entire mutation process was repeated 100,000 times to enable a rigorous search for peptides that produced the best RAPDF score (i.e., highest predicted stability).

### Computational design of novel inhibitory peptides

A 20 residue acid sliding window that moved from the N to the C terminus of the E protein in 10 residue acid increments was evaluated by a structural stability (pseudoenergy) optimization protocol using the RAPDF. A Metropolis Monte Carlo search algorithm [38] was used to change each amino acid in the 20 residue window to one of the other 19 naturally occurring amino acids, and the stability of corresponding peptide in the context of the entire E protein structure was evaluated. This process was iterated 100,000 times using RAPDF as the target scoring function. The Metropolis criterion was used to select a particular change in the simulation: if a particular change resulted in a better RAPDF score (lower pseudoenergy), then it was retained. If a particular change resulted in a worse RAPDF score (higher pseudoenergy), then a random choice, based on the score difference between the previous change and the current one, was made to retain the corresponding change. This procedure enables not only enables design of peptides that will result in high structural and binding stability (i.e., the best RAPDF scores/pseudoenergies), but also enables surmounting local minima encountered during the search. Computational optimization was performed on the four regions corresponding to the best RAPDF score, and therefore the highest binding potential, within the E protein as described above to generate variant peptides sequences.

### Viruses and cells

DENV-2 strain NG-C was obtained from R. Tesh at the University of Texas at Galveston. Virus was propagated in the *Macaca mulatta* kidney epithelial cell line, LLC-MK2 (ATCC catalog number CCL-7). Cells were grown in Dulbecco's modified eagle medium (DMEM) with 10% (v/v) fetal bovine serum (FBS), 2 mM Glutamax, 100 U/ml penicillin G, 100 µg/ml streptomycin and 0.25 µg/ml amphotericin B, at 37°C with 5% (v/v) CO_2_.

### Peptides

Peptides were synthesized by solid-phase N-α-9-flurenylmethyloxycarbonyl chemistry, purified by reverse-phase high performance liquid chromatography and confirmed by amino acid analysis and electrospray mass spectrometry (Genemed Synthesis, San Antonio, TX). Peptide stock solutions were prepared in 20% (v/v) dimethyl sulfoxide (DMSO): 80% (v/v) H_2_O, and concentrations determined by absorbance of aromatic side chains at 280 nm.

### Focus forming unit assay

LLC-MK2 target cells were seeded at a density of 1×10^5^ cells in each well of a 6-well plate 24 h prior to infection. Approximately 200 focus forming units (FFU) of virus were incubated with or without peptide in serum-free DMEM for 1 h at rt. Virus/peptide or virus/control mixtures were allowed to infect confluent target cell monolayers for 1 h at 37°C, with rocking every 15 m, after which time the medium was aspirated and overlaid with fresh DMEM/10% (v/v) FBS containing 0.85% (w/v) Sea-Plaque Agarose (Cambrex Bio Science, Rockland, ME) without rinsing. Cells with agar overlays were incubated at 4°C for 20 m to set the agar. Infected cells were then incubated at 37°C with 5% CO_2_ for 5 days. Infected cultures were fixed with 10% formalin overnight at 4°C, permeablized with 70% (v/v) ethanol for 20 m, and rinsed with phosphate buffered saline, pH 7.4 (PBS) prior to immunostaining. Virus foci were detected using a specific mouse mAb from hybridoma E60 (obtained from M. Diamond at Washington University), followed by horseradish peroxidase-conjugated goat anti-mouse immunoglobulin (Pierce, Rockford, IL), and developed using AEC chromogen substrate (Dako, Carpinteria, CA). Results are expressed as the average of at least two independent trials with three replicates each. IC_50_ values were determined using variable slope sigmoidal dose-response curve fits with GraphPad Prism 4.0 software (LaJolla, CA), except for DN81opt, which was determined graphically due to a lack of data points to produce a reasonable curve fit.

### Toxicity assay

Cytotoxicity of peptides was measured by monitoring mitochondrial reductase activity using the TACS™ MTT cell proliferation assay (R&D Systems, Inc., Minneapolis, MN) according to the manufacturer's instructions. Dilutions of peptides in serum-free DMEM were added to confluent monolayers of LLC-MK2 cells in 96-well plates for 1 h at 37°C, similar to the focus forming inhibition assays, and incubated at 37°C with 5% (v/v) CO_2_ for 24 h. Absorbance at 560 nm was measured using a Tecan GeniosPro plate reader (Tecan US, Durham, NC).

### Cryoelectron microscopy

DENV-2 NGC strain used for the cryoEM reconstructions was propagated in mosquito C6/36 cells. Virus was purified by precipitation with 40% PEG 8000 and then ultracentrifugation onto a 25% sucrose cushion. Virus was further purified by banding on a 10%–30% potassium tartrate gradient. The virus band was removed and dialyzed against 12 mM Tris pH 8.0, 120 mM NaCl, 1 mM EDTA, and concentrated using a Millipore Centricon filter. Purified virus was mixed with 1OAN or DN57opt at a concentration of 1 molecule of peptide for every E protein on the surface of the virus. The complex was incubated for half an hour at 37°C followed by half an hour at 4°C and then flash frozen on holey carbon grids in liquid ethane. Images of the frozen complex were taken with a Philips CM200 FEG transmission electron microscope (Philips, Eindhoven, The Netherlands) at a magnification 51,040 using an electron dose of approximately, 25e-/Å 2 using a Charge-Couple device.

### Peptide:E protein biolayer interferometry binding assay

Real time binding assays between peptides and purified DENV-2 S1 E protein were performed using biolayer interferometry on an Octet QK system (Fortebio, Menlo Park, CA). This system monitors interference of light reflected from the surface of a fiber optic sensor to measure the thickness of molecules bound to the sensor surface. Purified, recombinant, 80% truncated DENV-2 S1 E protein was obtained from Hawaii Biotechnology (Honolulu, HI). Peptides were N-terminally biotinylated with a 5∶1 molar ratio of NHS-LC-LC-Biotin (Pierce/ThermoFisher, Rockford, IL) in PBS pH 6.5 at 4°C. Excess biotinylation reagent was removed using Pepclean C-18 spin columns (Pierce/ThermoFisher, Rockford, IL). Biotinylated peptides were coupled to kinetics grade streptavidin high binding biosensors (Fortebio, Menlo Park, CA) at several different concentrations. Sensors coated with peptides were allowed to bind to E protein in PBS with 0.02% (v/v) Tween-20 and 1 mg/ml BSA at several different E protein concentrations. Binding kinetics were calculated using the Octet QK software package, which fit the observed binding curves to a 1∶1 binding model to calculate the association rate constants. E protein was allowed to dissociate by incubation of the sensors in PBS. Dissociation curves were fit to a 1∶1 model to calculate the dissociation rate constants. Binding affinities were calculated as the kinetic dissociation rate constant divided by the kinetic association rate constant.

### Post-infection treatment focus forming unit assay

Approximately 200 FFU of DENV-2 without peptide was allowed to bind and enter target cells for 1 h at 37°C as described for the focus forming unit assay. Unbound virus was then removed by rinsing with PBS and peptide was added to the cells for 1 h at 37°C. Cultures were washed again in PBS and agarose overlays, incubation, and immunological detection was conducted as described for the focus forming unit assay.

### Post-binding treatment focus forming unit assay

Approximately 200 FFU of DENV-2 were allowed to attach to cells for 45 min at 4°C, and then rinsed with cold PBS before peptide was incubated with the target cells for 45 min at 4°C. The cells were rinsed again with cold PBS, and agarose overlays, incubation, and immunological detection were conducted as described for the focus forming unit assay.

### Hemagglutination inhibition assay

Hemagglutination inhibition (HI) was performed according to [39] adapted to microtiter plates.

### Virus:cell binding inhibition assay

Binding inhibition assays were modified from Thaisomboonsuk, et al [40].}. Briefly, LLC-MK2 monolayers were rinsed in 4°C DMEM containing 0.8% BSA and 25 mM HEPES, pH 7.5. Virus was incubated at 4°C with peptides, control anti-dengue serum, or heparan sulfate in DMEM/BSA/HEPES for one hour before adding to the monolayers for 2 hours at 4°C. Monolayers were rinsed 3 times with cold DMEM/BSA/HEPES media prior to RNA extraction using the Qiagen RNeasy mini kit (Valencia, CA) per manufacturers instructions. Quantitative, real time, reverse transcriptase polymerase chain reaction (qRT-PCR) was conducted utilizing the Roche Lightcycler RNA Master SYBR Green 1 qRT-PCR kit (Basel, Switzerland), using primers Den_F (TTAGAGGAGACCCCTCCC) and Den_R (TCTCCTCTAACCTCTAGTCC) from Chutinimitkul et al [41].}. and the following cycling conditions: 1 h at 61°C, 30 s at 95°C, followed by 45 cycles of: 5 s at 95°C, 20 s at 61°C, and 30 s at 72°C. Cp values were used to estimate infectious units according to a standard curve. Independent assays were repeated three times, in duplicate or triplicate.

### Analysis

Graphs were generated using KaleidaGraph v.3.6 graphing software (Synergy Software, Reading, PA). Statistical analyses were performed using the GraphPad Prism 4.0 software package (GraphPad Software, San Diego, CA). P values less than 0.05 were considered significant.

## Results

### Computational optimization of hinge region peptide inhibitors

We had previously identified several E protein regions where peptides mimicking the E protein sequence might function as inhibitors. Several of these mimic peptides did not show substantial DENV inhibitory activity [9]. These included a peptide derived from the domain II fusion sequence (DN80, corresponding to amino acids 96–114 in the DENV-2 E protein) and two overlapping peptides derived from the domain II hinge region (DN57 and DN81, corresponding to amino acids 205–223 and 205–232, respectively). Predictions from crystal structures [11], [14], [15], as well as the previously confirmed inhibitory activity of an analogous WNV domain II hinge region peptide [9] lent support to the idea that the domain II hinge region was an attractive target for inhibition. Energy minimized peptides with sequences computationally optimized for structural stability and binding to the target regions, as evaluated by our residue-specific all-atom probability discriminatory function (RAPDF), were selected for further characterization and evaluation. These sequences generally had the best RAPDF scores (or “pseudoenergies”) for structural stability and binding, much better (lower) than the original wild type sequences (see Table 1 for original and optimized sequences.). These sequences, DN57opt, DN80opt and DN81opt, were selected for synthesis and evaluated for antiviral activity.

**Table 1:** Sequences and IC_50_ values of peptides.

Name	LOCATION	Sequence	IC_50_ (µM)
DN57wt	205–232	AWLVHTQWFLDLPLPWLPGADTQGSNWI	--*
DN57opt		RWMVWRHWFHRLRLPYNPGKNKQNQQWP	8±1
DN57opt-scram		RWRHLKKMQRLQPRNPNWPGQFWVHYNW	--
DN80wt	96–114	MVDRGWGNHAGLFGKGSIV	--*
DN80opt		MVIVQHQWMQIMRWPWQPE	--
DN81wt	205–223	AWLVHRQWFLDLPLPWLPG	--*
DN81opt		RQMRAWGQDYQHGGMGYSC	36±6
1OAN1wt	41–60	LDFELIKTEAKQPATLRKYC	ND
1OAN1		FWFTLIKTQAKQPARYRRFC	7±4
1OAN1-scram		QQCFRFPALRKKATYTRFWI	--
1OAN2wt	131–150	QPENLEYTVVITPHSGEEHA	ND
1OAN2		YPENLEYRVYITPHPGEEHH	--
1OAN3wt	251–270	VVLGSQEGAMHTALTGATEI	ND
1OAN3		EWSKHREGRWHTALTGATEI	--
1OAN4wt	351–370	LITVNPIVTEKDSPVNIEAE	ND
1OAN4		WHTVEPIVTEKDRPVNYEWE	--

Names and sequences for previously tested wild type peptides are denoted with an asterisk [9], computationally designed peptides, wild type sequence peptides, and scrambled control peptides are shown. 50% inhibitory concentrations (IC_50_ values ± sem) determined from sigmoidal curve fits to the dose response curves in Figure 2 are given for DN57opt, DN81opt, and 1OAN1. Tested peptides that did not achieve 50% inhibition are noted with a dashed line. Peptides that were not tested for antiviral activity are noted ND (no data).

### Computational design and optimization of novel peptide inhibitors

**Figure 1:**
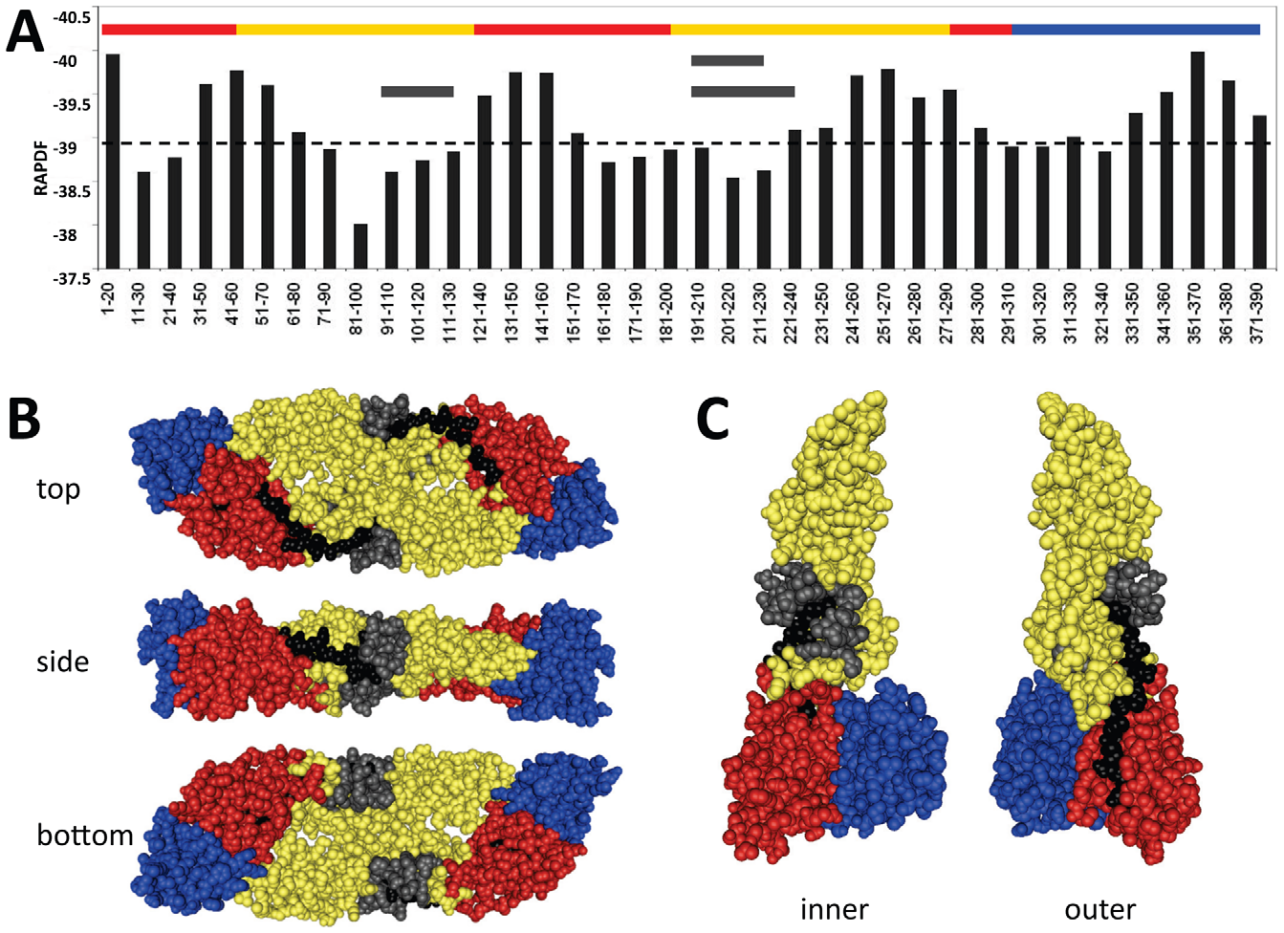
Locations of predicted peptides on the DENV-2 E protein primary sequence. (A) The DENV-2 E protein is shown linearly from N to C terminus. The three domains are color coded above, domain I is shown in red, domain II is yellow, and domain III is in blue according to [46]. The calculated RAPDF scores of a sliding window of 20 amino acid peptide sequences are shown graphically by vertical black lines. Four major high binding regions are predicted, from amino acids 31–70, 121–160, 241–270, and 341–380, respectively, corresponding to the locations of peptides 1OAN1, 1OAN2, 1OAN3, and 1OAN4. The locations of the other optimized peptides are indicated by three horizontal black lines, amino acids 96–114 is DN80opt, 205–223 is DN81opt, and 205–232 is DN57opt. (B) Structure of dimeric dengue E protein in the pre-fusion conformation showing locations of inhibitory peptides. Top, side, and bottom views are shown. Structures are color coded as above. Black and grey residues show the positions of the 1OAN1 and DN57opt peptides respectively. (C) Structure of monomeric dengue E protein in the low pH post-fusion conformation. Inner (interacting) and outer surfaces are shown.

To identify additional novel peptide inhibitors and their corresponding targets, a 20 residue sliding window that moved from the N to the C terminus of the DENV-2 strain S1 E protein (PDB ID 1OAN) in 10 residue acid increments was evaluated by a structural stability (pseudoenergy) optimization protocol using the RAPDF. A Metropolis Monte Carlo search algorithm [38] was used to change each amino acid in the 20 residue window to one of the 19 other naturally occurring amino acids, and the stability of each corresponding peptide in the context of the entire E protein structure was evaluated. Our approach identified four E protein regions with the potential for the highest *in situ* binding affinities. These correspond to DENV-2 strain S1 E protein amino acids 41–60, 131–150, 251–270, and 351–370 (see Figure 1) that were selected for synthesis and antiviral testing (1OAN1, 1OAN2, 1OAN3, and 1OAN4).

### Inhibition of DENV-2

**Figure 2:**
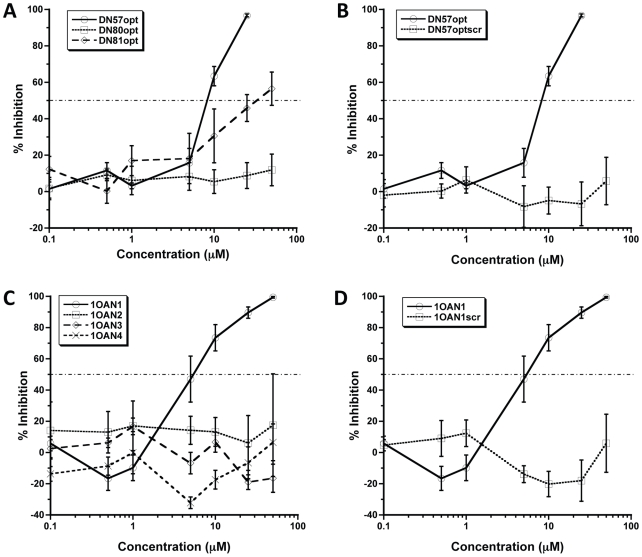
Inhibition of DENV-2 *in vitro*. Increasing concentrations of optimized inhibitor peptides and corresponding scrambled peptides of identical composition were tested against DENV-2 in a focus forming unit reduction assay. (A) Optimized peptides (B) DN57opt and corresponding scrambled peptide of identical composition (C) Novel peptides (D) 1OAN1 and corresponding scrambled peptide of identical composition. Error bars are ±sem.

In order to verify the effectiveness of the binding optimization process and peptide design, synthesized peptides were tested for antiviral activity against DENV-2 strain NG-C in a focus forming unit (FFU) reduction assay. DENV-2 strains S1 (GenBank accession number M19197.1) and NG-C (GenBank accession number AF038403.1) share 98% amino acid sequence identity in the E protein and the majority of differences are conservative. Dose response curves generated for the optimized peptides DN57opt, DN80opt, and DN81opt are shown in Figure 2A. The domain II region peptides, DN57opt and DN81opt displayed IC_50_ values of 8±1 µM and 36±6 µM (mean ± sem) respectively, while no inhibition of infection was observed with the fusion region peptide, DN80opt. Correspondingly, maximum inhibition of 97% and 57% was achieved at 20 µM and 50 µM for DN57opt and DN81opt. Both DN57opt and DN81opt showed improved inhibition of DENV-2 compared to their non-optimized counterparts, with DN57opt and DN81opt showing a nearly 14 fold and a 2 fold increase, respectively, in inhibition of DENV-2 at equivalent concentrations [9]. The most active inhibitor, DN57opt was chosen for further study. A scrambled version of DN57opt (DN57optscr) did not display inhibition at any concentration tested (Figure 2B). Four *de novo* designed peptides, 1OAN1, 1OAN2, 1OAN3, and 1OAN4 were also tested for inhibitory activity using the same FFU reduction assay (Figure 2C). 1OAN1 was found to be an effective inhibitor of DENV-2 infection with an IC_50_ of 7±4 µM and a maximum inhibition of 99% at 50 µM. A scrambled version of 1OAN1 (1OAN1scr) did not inhibit infection by DENV-2 at any concentration tested (Figure 2D). In addition to these full dose response inhibition experiments using approximately 100 infectious units of virus, both the DN57opt and 1OAN1 peptides were also capable of inhibiting 4,000 infectious units of virus (data not shown).

### Peptide toxicity

**Figure 3:**
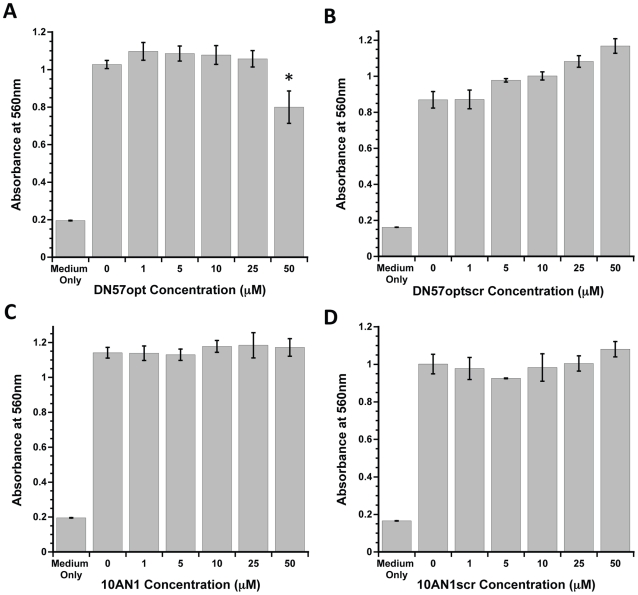
Inhibitory peptide toxicity *in vitro*. Increasing concentrations of peptides were tested in an MTT mitochondrial reductase activity assay. Error bars are ± sd. (A) DN57opt (B) Scrambled version of DN57opt (C) 1OAN1 (D) Scrambled version of 1OAN1. * denotes a statistically significant difference from the no peptide control.

Because toxicity could result in a decrease in focus formation and be interpreted as evidence of antiviral activity, the inhibitory peptides and their scrambled versions were assessed for cellular toxicity. Confluent monolayers of LLC-MK2 cells used in FFU reduction assays were exposed to increasing concentrations of peptide before measuring mitochondrial reductase activity using an MTT mitochondrial reductase activity assay (Figure 3). When we initially performed these assays to exactly mimic the focus forming unit assay by waiting five days after peptide exposure, we saw no evidence of toxicity at any concentration of any peptide (data not shown). However, we found that a shorter post-exposure incubation time revealed a subtle toxicity on the part of one of the peptides. Apparently, waiting more than 24 h post-exposure gives the cells a chance to recover and conceals this effect. At 24 h post-exposure, DN57opt was found to be mildly toxic to cells at 40 µM (one-way ANOVA with Dunnet's *post hoc* test, P = 0.0004, N = 18), so only inhibitory data using lower, nontoxic concentrations was considered. Peptides DN57optscr, 1OAN1, and 1OAN1scr were not toxic at any concentration tested (one-way ANOVA, P>0.05).

### DN57opt and 1OAN1 cause changes to the surface of DENV-2 virus

**Figure 4:**
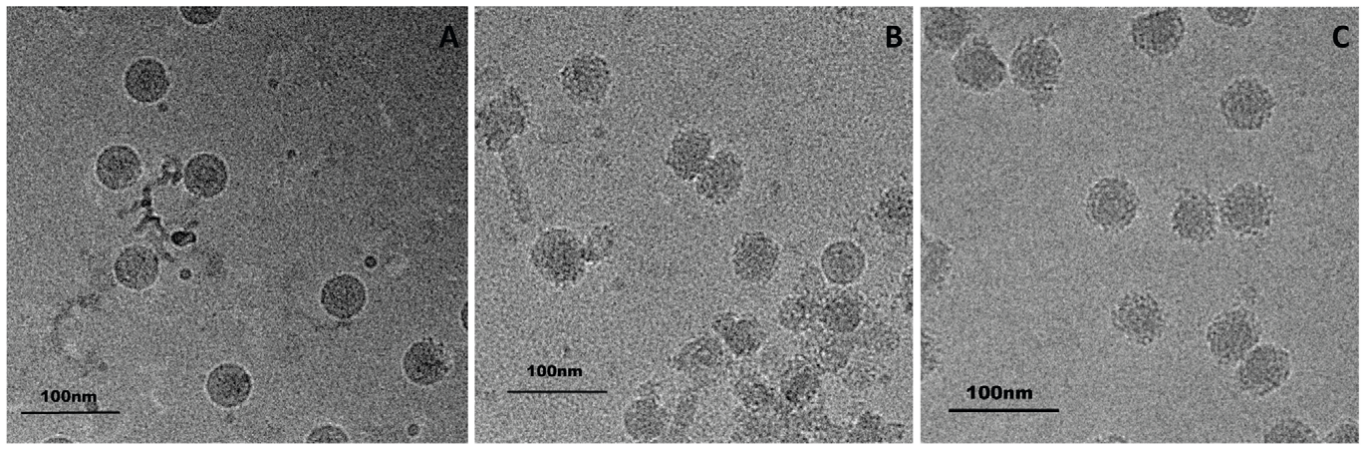
Cryoelectron microscopy. Purified and concentrated virus was prepared with or without incubation with peptides and then flash frozen for imaging. Panels show (A) virus only, (B) virus incubated with DN57opt, (C) virus incubated with 1OAN1. Scale bars indicate 100 nm.

Cryoelectron microscopy (cryoEM) was used to visualize the effect of the DN57opt and 1OAN1 peptides on DENV-2 viral particles. Control dengue virions exhibited the normal, nearly smooth outer surface typical of mature flaviviruses [42]. The surfaces of the virus particles werebecome followingrough after treatment with peptides, implying a possible rearrangement of the envelope glycoproteins (Figure 4). The treated virions no longer showed icosohedral symmetry, Attempts to reconstruct the structure of virus complexed with DN57opt and 1OAN1 structures by imposing icosahedral symmetry failed, indicating the viruses are no longer icosahedral. Control treatments with equivalent DMSO alone did not produce this morphological alteration.

### DN57opt and 1OAN1 bind to soluble DENV-2 E protein

**Figure 5:**
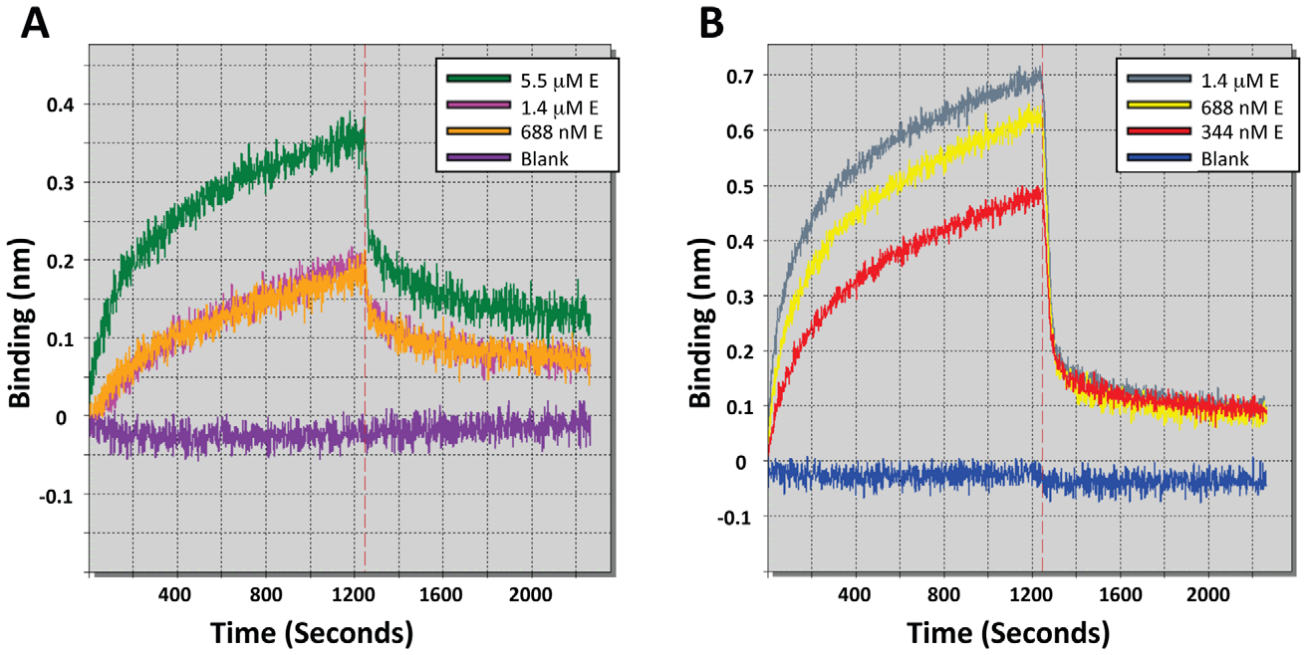
Peptide:E protein binding assay. Biolayer interferometry was used to assay the binding of the peptides to truncated E Protein. The association and dissociation of increasing concentrations of truncated E protein to peptides DN57opt (A) and 1OAN1 (B) are shown. A buffer blank (PBS, 0.02% Tween-20, 0.1% BSA) containing no E protein was run for each peptide. The affinity of the peptides for the truncated E protein was calculated (DN57opt K_D_ = 1.2×10^−6^±0.6×10^−6^ M (mean±sd), 1OAN1 K_D_ = 4.5×10^−7^±2.0×10^−7^ M).

Biolayer interferometry was performed to examine binding of the peptides to purified, truncated DENV-2 E protein. Amino terminally biotinylated peptides were immobilized onto streptavidin biosensors and then the association and dissociation of truncated E protein with the immobilized peptides was monitored. The interactions of three different concentrations of truncated E protein to peptides DN57opt and 1OAN1 are shown (Figure 5). A buffer blank containing no E protein was run for each peptide. The affinities of the peptides for the truncated E protein were calculated with a 1∶1 binding model: DN57opt K_D_ = 1.2×10^−6^±0.6×10^−6^ M (mean±sd), 1OAN1 K_D_ = 4.5×10^−7^±2.0×10^−7^ M. While the data for the 1OAN1 peptide show a lower K_D_, these numbers are not statistically different (unpaired student's T-test, P = 0.16, N = 3). The association rate constants were: DN57opt k_a_ = 8.0×10^2^±5.0×10^2^ M^−1^s^−1^, 1OAN1 k_a_ = 3.9×10^3^±1.5×10^3^ M^−1^s^−1^. The dissociation rate constants were: DN57opt k_d_ = 7.7×10^−4^±1.7×10^−4^ s^−1^, 1OAN1 k_d_ = 1.6×10^−3^±0.2×10^−3^ s^−1^. We have previously used this system to characterize the binding affinities of several human monoclonal antibodies for DENV E proteins [43].

### Treatment of cells with DN57opt and 1OAN1 post-infection does not inhibit replication of DENV-2

**Figure 6:**
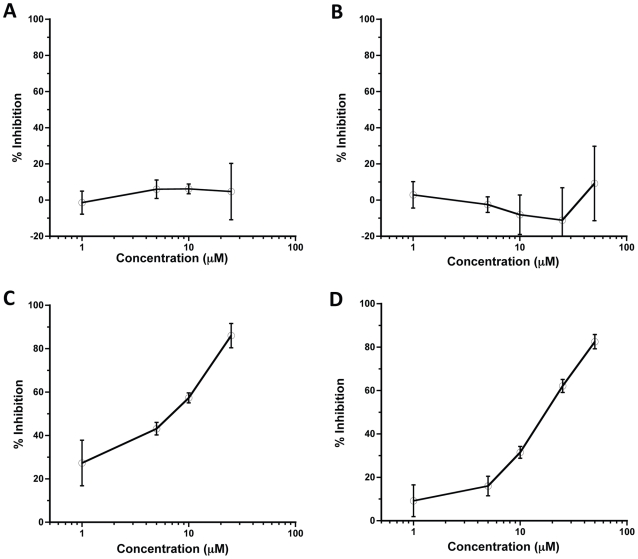
Post-infection and post-binding peptide treatments. Treatment of cells with increasing concentrations of (A) DN57opt and (B) 1OAN1 after DENV-2 has infected cells shows no significant inhibition. Treatment with (C) DN57opt or (D) 1OAN1 after DENV-2 has bound to LLCMK-2 cells at 4°C for one hour inhibits infection. Error bars are ±sem.

In order to determine if the peptides were exerting their effects on post-entry steps in the virus replication cycle, DENV-2 was allowed to infect LLC-MK2 cells before peptide was added to the cells (Figure 6). No inhibition of viral replication was observed at any concentration of DN57opt (Figure 6A) or 1OAN1 in these assays (Figure 6B), indicating that the peptides are not acting at a post-infection step.

### Treatment of cells with DN57opt and 1OAN1 after virus binding to cells but before entry inhibits DENV-2 infection

Since we had determined that inhibition with both peptides occurs at a viral entry step, we asked if infection could still be inhibited after virus had bound to the surface of target cells. We bound virus to cells at 4°C, then treated with increasing concentrations of DN57opt or 1OAN1 before warming the cells back to 37°C and allowing the infections to progress (Figure 6C and D). Inhibition of viral entry was observed for both peptides when added to the virus after it was bound to target cells.

### DN57opt and 1OAN1 block virus binding to target cells

**Figure 7:**
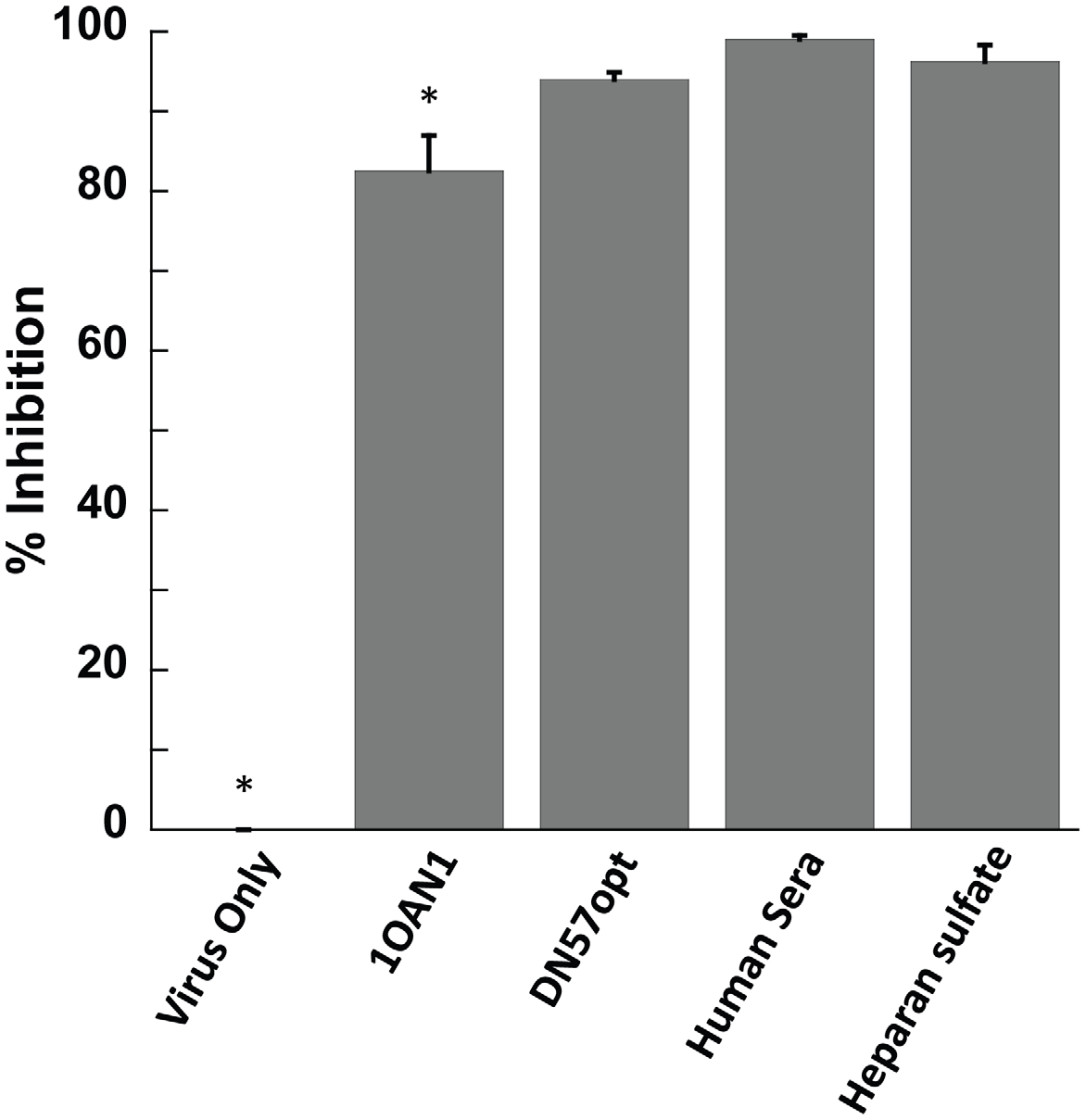
Quantitative reverse transcriptase PCR virus:cell binding. Virus pre-incubated with either DN57opt or 1OAN1 shows reduced binding to cells compared to control virus without peptide. Pre-incubation of virus with pooled human anti-dengue serum or heparan sulfate similarly shows reduced cell binding. * Indicates a significant difference (p<0.05) from all others by 1-way ANOVA followed by Tukey's posthoc test.

To determine if the peptides interefere with virus:cell interactions, we conducted two different experiments. We first performed hemagglutination inhibition assays, but were unable to detect any inhibition of the ability of viral antigen to agglutinate red blood cells (data not shown). To further investigate virus:cell binding in a more relevant system, we treated virus with DN57opt or 1OAN1, bound the virus to cells, and washed the cells repeatedly at 4°C before measuring the amount of virus remaining on the cells by quantitative rt-PCR. Both peptides showed evidence of ability to block virus:cell binding compared to control virus without peptide (Figure 7). Treatment of virus with pooled human anti-dengue serum or heparan sulfate similarly showed reduced cell binding.

## Discussion

We have used computational methods to design multiple peptide inhibitors of the DENV E glycoprotein. Importantly, out of seven peptides synthesized and tested, two peptides with high activity and one peptide with intermediate activity were identified. A high resolution crystal structure of the pre-fusion conformation of the DENV-2 E [14] was used as the starting point to generate *in situ* energy minimized peptides. Two distinct approaches were used for the design of these peptides. First, we built upon previous work targeting DENV fusion peptide and domain II hinge regions with naturally occurring E protein sequences from these regions [9]. No inhibitory activity was found for the optimized fusion peptide region sequence (DN80opt), indicating that this region may not be a promising target mechanistically for DENV peptide inhibitors. Since an analogous, naturally occurring WNV domain II hinge region peptide was shown to be inhibitory against WNV [9], we reasoned that a more tightly binding analog of this region in the DENV E protein could be designed and might have improved inhibitory activity. This turned out to be correct, and we identified two distinct binding-optimized peptide sequences to this region with antiviral activity, DN57opt and DN81opt. This supports previous predictions of hinge region inhibitors and the proposed mechanism of fusion based on hinge region movements [11], [14], [15]. The second approach to designing peptide inhibitors was to identify peptides with non-native sequences derived from E protein regions that are highly stable in terms of structure and binding as evaluated by an all-atom scoring function (RAPDF). This identified four regions that were used to derive additional optimized peptides (Figure 1). Of the four resulting peptides tested, one, 1OAN1, was identified as having antiviral activity. This confirms the use of the sliding window RAPDF minimization approach for finding tightly binding protein ligands [23], [24]. It is perhaps not surprising that computational binding optimization increased the activity of previously inactive peptides that were based on naturally occurring E protein sequences. Naturally occurring sequences have multiple balancing selection pressures that may limit their binding stability *in vivo*. The combined use of primary sequence prediction tools [9] and structural optimization tools [23], [24] should be a valuable approach for finding binding partners and inhibitors for other protein targets.

Neither peptide showed inhibitory activity when added directly to cells after infection had already occurred, indicating that the peptides were acting during an entry step in the virus life cycle, and sequence scrambled versions of the two most active peptides were inactive, confirming sequence specific activity. Both peptides also block virus:cell binding, but are still capable of inhibiting infection even when added after virions have already bound to the surface of target cells.

CryoEM was used to visualize the effect of the peptides on DENV-2 virions. The surface of virions appeared to change from smooth to rough after incubation with the antiviral peptides. This suggests that there may be an alteration of the arrangement of the surface envelope protein (Figure 4). Biolayer interferometry was used to measure the kinetics of binding between the peptides and soluble, truncated E protein (Figure 5). These binding studies showed a direct interaction between the peptides and DENV-2 E protein with affinities in the 1 µM range and relatively fast on/off rates. The cryoEM images demonstrate that these inhibitory peptides probably cause structural deformations in intact viral particles, but do not provide information about the kinetics of these changes. It is possible that the peptides trap the viral E proteins in some conformation that is part of the normal breathing of the viral particles, and that this interferes with cell binding and entry.

The DN57opt and 1OAN1 peptides were designed for optimized binding to the pre-fusion E structure and we show direct evidence for this interaction, both with the purified, monomeric E protein, and with virion particles. These peptides likely function by displacing portions of the E protein and interfering with normal cell binding or the structural changes during entry. Although separate in the primary protein sequence, the regions targeted in the design the DN57 and 1OAN1 peptides are partially adjacent to each other in the crystal structure, with the C terminus of the 1OAN1 region occupying a pocket surrounded by the DN57 region (See Figure 1). We stress that we do not know the structures of the bound and inhibited peptide/E protein complexes, but these structures may shed light on the mechanistic details of cell binding and fusion. Taken together, our results support the hypothesis that both of these peptides interact directly with DENV-2 E proteins and are entry inhibitors.

Despite difficulties with oral administration and degradation in the digestive tract, peptides may make useful antiviral agents when targeted against viral envelope proteins. Directing inhibitors to viral surface proteins avoids the major difficulty of crossing cellular membranes in order to reach the target. For example, peptide inhibitors of intercellular viral targets, such as proteases or polymerases, would need to cross the cell plasma membrane, and in the case of flaviviruses, possibly internal membrane bound replication and assembly compartments. The HIV entry inhibitor T-20 (Fuzeon) is a peptide, and in the context of a chronic infection, repeated life-long injections are problematic. DENV is an acute infection and most severe DENV infections require intravenous fluid support, facilitating delivery of anti-DENV peptides by this route.

We have established the existence of multiple, distinct inhibitory peptides targeting the DENV E glycoprotein and confirmed the utility of rational design using structural data for developing DENV E protein inhibitors. Applications of this strategy should also be possible for the generation and refinement of lead compounds for other viral envelope fusion proteins. It would be optimistic to propose that any single antiviral would provide an effective treatment for DENV given the enormous genetic variability of the four serotypes and multiple substrains. Different classes of inhibitors targeting the E protein and other DENV targets [44], [45], could form the basis for the development of a combination treatment plan to combat this disease.

## Supporting Information

Alternative Language Abstract S1 Translation of the abstract into Thai by Ekachai Jenwitheesuk.
(0.03 MB DOC)

Alternative Language Abstract S2 Translation of the abstract into Spanish by Sharon Isern.
(0.04 MB DOC)
