# Supplementary material for: Structural Optimization and De Novo Design of Dengue Virus Entry Inhibitory Peptides
Source: PLoS Negl Trop Dis. 2010 Jun 22;4(6):e721. doi: 10.1371/journal.pntd.0000721 (PMC2889824; doi:10.1371/journal.pntd.0000721)
Supplement: Alternative Language Abstract S1 — Translation of the abstract into Thai by Ekachai Jenwitheesuk. (0.03 MB DOC) [file pntd.0000721.s001.doc]

**ABSTRACT**

Viral fusogenic envelope proteins are important targets for the development of inhibitors of viral entry. We report an approach for the computational design of peptide inhibitors of the dengue 2 virus (DENV-2) envelope (E) protein using high-resolution structural data from a pre-entry dimeric form of the protein. By using predictive strategies together with computational optimization of binding “pseudoenergies”, we were able to design multiple peptide sequences that showed low micromolar viral entry inhibitory activity. The two most active peptides, DN57opt and 1OAN1, were designed to displace regions in the domain II hinge, and the first domain I/domain II beta sheet connection, respectively, and show fifty percent inhibitory concentrations of 8 and 7 uM respectively in a focus forming unit assay. The antiviral peptides were shown to interfere with virus:cell binding, interact directly with the E proteins and also cause changes to the viral surface using biolayer interferometry and cryo-electron microscopy, respectively. These peptides may be useful for characterization of intermediate states in the membrane fusion process, investigation of DENV receptor molecules, and as lead compounds for drug discovery.

Translated by Ekachai Jenwitheesuk

**บทคัดย่อ**

โปรตีนเอนวีลอปเป็นโปรตีนเป้าหมายที่สำคัญในการพัฒนายาต้านไวรัสเพื่อป้องกันไม่ให้ไวรัสเข้าสู่เซล งานวิจัยนี้ใช้วิธีการทางคอมพิวเตอร์ออกแบบสารยับยั้งชนิดเปปไตด์ (peptide inhibitors) เพื่อยับยั้งการทำงานของโปรตีนเอนวีลอปของไวรัสเด็งกี่ซีโรทัยป์ 2 โดยใช้โครงสร้างสามมิติที่มีความละเอียดสูงของโปรตีนเอนวีลอปของไวรัสเด็งกี่ซึ่งอยู่ในรูป pre-entry dimeric เป็นต้นแบบในการคำนวณ “ค่าพลังงานเทียม” (pseudoenergies) เพื่อค้นหาลำดับกรดอะมิโนของเปปไตด์ที่เหมาะสม จากการคำนวณ พบเปปไตด์สองเส้นที่มีประสิทธิภาพในการยับยั้งไวรัสเด็งกี่ได้ที่ความเข้มข้นระดับไมโครโมลาร์ ได้แก่ เปปไตด์ DN57opt และเปปไตด์ 1OAN1 ซึ่งถูกออกแบบมาเพื่อให้เข้าไปแทนที่ส่วนจุดหมุนของโดเมนที่สอง และส่วนเบต้าชีตที่เชื่อมระหว่างโดเมนที่หนึ่งและโดเมนที่สองของโปรตีนเอนวีลอป ตามลำดับ โดยมีค่า fifty percent inhibitory concentrations เท่ากับ 8 ไมโครโมลาร์ สำหรับเปปไตด์ DN57opt และ 7 ไมโครโมลาร์ สำหรับเปปไตด์ 1OAN1 เมื่อศึกษาโดยใช้เครื่อง biolayer interferometry และ cryo-electron microscopy พบว่าเปปไตด์ทั้งสองนี้ ทำปฏิกิริยาโดยตรงกับโปรตีนเอนวีลอปของไวรัส โดยสามารถรบกวนไม่ให้ไวรัสจับกับเซลเป้าหมาย และทำให้ผิวเปลือกนอกของไวรัสเปลี่ยนแปลงไป ตัวยับยั้งชนิดเปปไตด์ที่ได้จากงานวิจัยนี้อาจมีประโยชน์ในการศึกษากระบวนการหลอมรวมเยื่อหุ้มเซล (membrane fusion) การศึกษาโมเลกุลของรีเซ็บเตอร์ (receptor) ของไวรัสเด็งกี่ และใช้เป็นสารประกอบตั้งต้นในการค้นหายา
