# Supplementary material for: Structural Optimization and De Novo Design of Dengue Virus Entry Inhibitory Peptides
Source: PLoS Negl Trop Dis. 2010 Jun 22;4(6):e721. doi: 10.1371/journal.pntd.0000721 (PMC2889824; doi:10.1371/journal.pntd.0000721)
Supplement: Alternative Language Abstract S2 — Translation of the abstract into Spanish by Sharon Isern. (0.04 MB DOC) [file pntd.0000721.s002.doc]

**ABSTRACT**           Viral fusogenic envelope proteins are important targets for the development of inhibitors of viral entry. We report an approach for the computational design of peptide inhibitors of the dengue 2 virus (DENV-2) envelope (E) protein using high-resolution structural data from a pre-entry dimeric form of the protein. By using predictive strategies together with computational optimization of binding “pseudoenergies”, we were able to design multiple peptide sequences that showed low micromolar viral entry inhibitory activity. The two most active peptides, DN57opt and 1OAN1, were designed to displace regions in the domain II hinge, and the first domain I/domain II beta sheet connection, respectively, and show fifty percent inhibitory concentrations of 8 and 7 μM respectively in a focus forming unit assay. The antiviral peptides were shown to interfere with virus:cell binding, interact directly with the E proteins and also cause changes to the viral surface using biolayer interferometry and cryo-electron microscopy, respectively. These peptides may be useful for characterization of intermediate states in the membrane fusion process, investigation of DENV receptor molecules, and as lead compounds for drug discovery.

Translated by Sharon Isern

Proteínas fusogénicas de la envoltura vírica son objetivos importantes para el desarrollo de inhibidores de la entrada del virus. Se presenta un enfoque para el diseño computacional de los inhibidores péptidos de la proteína de la envoltura (E) del virus del dengue 2 (DENV-2) utilizando datos de alta resolución estructural de la forma dimérica de la proteína de pre-entrada. Mediante el uso de estrategias de predicción, junto con la optimización computacional de las "pseudoenergías" de la unión entre los péptidos y la proteína, hemos sido capaces de diseñar secuencias peptídicas múltiples que mostraron actividad micromolar baja contra el virus. Los dos péptidos más activos, DN57opt y 1OAN1, fueron diseñados para desplazar la región bisagra en el dominio II, y la primera conexión de lámina beta entre el dominio I y el dominio II, respectivamente, muestran concentraciones inhibitorias del cincuenta por ciento de 8 y 7 μM, respectivamente en un ensayo de unidad de formación de foco. Se ha demostrado que los péptidos antivirales fueron capaces de interferir con la unión del virus a la célula, interactuar directamente con las proteínas E y también causar cambios en la superficie viral usando interferometría de biocapa y crio-microscopía electrónica, respectivamente. Estos péptidos pueden ser útiles para la caracterización de estados intermedios en el proceso de fusión de la membrana, la investigación de las moléculas receptoras DENV y como compuestos principales para el descubrimiento de fármacos.
